# Psychological Well-Being of Parents of Very Young Children With Type 1 Diabetes – Baseline Assessment

**DOI:** 10.3389/fendo.2021.721028

**Published:** 2021-08-12

**Authors:** Carine de Beaufort, Ineke M. Pit-ten Cate, Ulrike Schierloh, Nathan Cohen, Charlotte K. Boughton, Martin Tauschmann, Janet M. Allen, Katrin Nagl, Maria Fritsch, James Yong, Emily Metcalfe, Dominique Schaeffer, Muriel Fichelle, Alena G. Thiele, Daniela Abt, Kerstin Faninger, Julia K. Mader, Sonja Slegtenhorst, Nicole Ashcroft, Malgorzata E. Wilinska, Judy Sibayan, Craig Kollman, Sabine E. Hofer, Elke Fröhlich-Reiterer, Thomas M. Kapellen, Carlo L. Acerini, Fiona Campbell, Birgit Rami-Merhar, Roman Hovorka

**Affiliations:** ^1^Diabetes & Endocrine Care Clinique Pediatrique (DECCP)-Pediatric Clinic, Pediatric Clinic/Centre Hospitalier de Luxembourg DECCP, Luxembourg, Luxembourg; ^2^Department of Pediatrics, University Clinic Brussels, Brussels, Belgium; ^3^Luxembourg Center for Educational Assessment, University of Luxembourg, Esch sur Alzette, Luxembourg; ^4^Jaeb Center for Health Research, Tampa, FL, United States; ^5^Wellcome Trust-Medical Research Council (MRC) Institute of Metabolic Science, University of Cambridge, Cambridge, United Kingdom; ^6^Department of Pediatrics, University of Cambridge, Cambridge, United Kingdom; ^7^Department of Pediatric and Adolescent Medicine, Medical University of Vienna, Vienna, Austria; ^8^Department of Paediatrics and Adolescent Medicine, Medical University of Graz, Graz, Austria; ^9^Department of Paediatric Diabetes, Leeds Children´s Hospital, Leeds, United Kingdom; ^10^Division of Paediatric Diabetology, University of Leipzig, Leipzig, Germany; ^11^Department of Pediatrics 1, Medical University of Innsbruck, Innsbruck, Austria; ^12^Department of Internal Medicine, Division of Endocrinology and Diabetology, Medical University of Graz, Graz, Austria; ^13^Department of Nutrition & Dietetics, Cambridge University Hospitals National Health Service (NHS) Foundation Trust, Cambridge, United Kingdom

**Keywords:** hypoglycemia fear, parental well-being, very young children, type 1 diabetes, child behavior, sleepiness

## Abstract

**Background:**

Type 1 diabetes in young children is a heavy parental burden. As part of pilot phase of the KIDSAP01 study, we conducted a baseline assessment in parents to study the association between hypoglycemia fear, parental well-being and child behavior.

**Methods:**

All parents were invited to fill in baseline questionnaires: hypoglycemia fear survey (HFS), WHO-5, Epworth Sleepiness Scale and Strength and Difficulties Questionnaire (SDQ).

**Results:**

24 children (median age: 5-year, range 1-7 years, 63% male, mean diabetes duration: 3 ± 1.7 years) participated. 23/24 parents filled out the questionnaires. We found a higher score for the hypoglycemia fear behavior 33.9 ± 5.6 compared to hypoglycemia worry 34.6 ± 12.2. Median WHO-5 score was 16 (8 - 22) with poor well-being in two parents. Median daytime sleepiness score was high in five parents (>10). For six children a high total behavioral difficulty score (>16) was reported. Pro social behavior score was lower than normal in six children (<6). Parental well-being was negatively associated with HFS total (*r* = - 0.50, *p* <.05) and subscale scores (*r* = - 0.44, *p* <.05 for HFS-Worry and HFS-Behavior), child behavior (*r* = - 0.45, *p* = .05) and positively with child age and diabetes duration (*r* = 0.58, *p* <.01, *r* = 0.6, *p* <.01). HFS, parental well-being nor daytime sleepiness are associated with the HbA1c.

**Conclusion:**

Regular screening of parental well-being, hypoglycemia fear and child behavior should be part of routine care to target early intervention.

## Introduction

International recommendations propose a HbA1c of <7.0% (<53 mmol/mol) in persons with type 1 diabetes (T1D) to prevent acute and late complications (1). Achieving near normal glucose values requires a continuous adjustment of insulin treatment around food intake, daily activities and hormonal changes. In preschool children these choices are determined by the parent/caregiver and pose continuous challenges and stress (2).

Especially the fear of hypoglycemia has been associated with increased stress in parents of younger children (3–5). Fear of hypoglycemia can lead to frequent nocturnal blood glucose measurements and subsequent sleep deprivation (6), which in turn may affect parents´ well-being as well as the (perceived) behavior of their child (7, 8). New technology such as insulin or sensor-augmented pumps should improve metabolic control. Better control may reduce (perceived) child behavioral problems (9) and parental anxiety around high and low blood glucose excursions (7, 10). Regardless of treatment regimen, parents/caregivers of young children with T1D often report their anxiety caused by glucose fluctuations during the night (11, 12). This suggests that these technologies, although providing improved metabolic outcome, may not yet alleviate parental stress levels.

Next steps in technology advancement include the use of the artificial pancreas, where different algorithms are used to steer insulin administration (13). An initial evaluation of the use of the Cambridge algorithm in toddlers demonstrated the feasibility of hybrid closed-loop insulin delivery in young children with diabetes (14), whereby parents reported reduced diabetes management burden and improved sleep quality (15). The current paper presents data from this baseline assessment with a specific focus on parental well-being in relation to fear of hypoglycemia.

## Methods

The KIDSAP01 study (NCT03101865) aims to evaluate a hybrid closed-loop insulin administration system, based on the Cambridge algorithm, under free-living conditions in very young children with T1D. A feasibility study was conducted to pretest the setup of the large-scale trial and to determine the specific needs of young children with T1D and their parents. In regards to the latter, parents of children participating in study completed questionnaires on hypoglycemia fear, parental well-being, daytime sleepiness and child behavior at baseline.

### Participants

The patient population has been described in detail previously (14, 15). To summarize, 24 children (median age 5 years, range 1-7 years, 63% male) with T1D from four countries (UK, Luxembourg, Germany and Austria) and seven clinics were included. Mean diabetes duration was 3.0 ± 1.7 years (range 0.5-6.4 years). All children were treated for at least three months with insulin pumps. The majority of children (n=18) used the Medtronic 640G pumps, 4 had a Medtronic 554 pump, 1 used an Animas Vibe and 1 a Roche Insight. The Medtronic Guardian REAL time sensor was used by 15 children, the ABBOTT LIBRE was used by 4 children and 3 children did not wear a sensor at baseline of this study.

HbA1c levels at baseline were used as an indicator of metabolic control (mean HbA1c, 7.4 ± 0.7% (57 ± 8 mmol/mol) and range 5.9-8.7% (41 - 72 mmol/mol). In the three months prior to the study, no severe hypoglycemia nor diabetic ketoacidosis had been observed. Twenty-three of the 24 parents filled out the questionnaire at the start of the study.

### Measures

The Hypoglycemia Fear Survey (HFS) for Parents (16) is a 26-item survey comprised of a HFS-worry and a HFS-behavior subscale. All items are scored on a 5-point Likert scale (1 = “never”; 5 = “always”). Higher scores reflect higher hypoglycemia fear.

The WHO-5 questionnaire has been extensively used to assess well-being in persons with diabetes and their family (17). It includes five items, with item scores ranging from 0 to 5. Scores under 13 are indicative of poor well-being and a score of below 7 warrants further testing for depression (18).

The Epworth Sleepiness scale evaluates sleepiness and risk to doze off during the day (19). It includes eight questions, scored between 0 and 3, whereby total scores > 9 indicate excessive sleepiness.

The strength and difficulties questionnaire is a screening tool to identify psychosocial problems in (pre-)school children (20–22). It includes 25 items comprising four subscales measuring difficulties (emotional, conduct or peer problems and hyperactivity), and a pro-social subscale rating the strengths. The total score is based on the first 4 subscales, whereby a total score > 15 is indicative of a high risk for psychosocial problems (23).

### Statistical Analyses

First descriptive data will be reviewed to identify the percentage of parents scoring within (ab)normal ranges on the standardized measures. In addition, correlational analyses will be conducted to identify relationships between indicators of parental well-being and child metabolic control and behavior. Based on previous research we expected positive relationships between parental well-being and metabolic control and child behavior. Given the exploratory nature of the pilot study, no a-priori power analyses were conducted.

## Results

Descriptive results are provided in **Table 1**. Results suggest that parents´ fear of hypoglycemia is signified by their behavior rather than their worries. Most pronounced fear-related behaviors reflected parents making sure their child had access to fast-acting carbohydrates and getting up in the middle of the night to check the child´s blood sugar levels. High mean worry scores were reported for low glucose levels whilst child was sleeping and for parent not being around when the child has low blood sugar. Poor well-being was reported by 2/23 (9%) parents, whereas 5/23 (22%) parents scored low on the question ‘I woke up feeling fresh and rested’. This question also generated the lowest mean score for the sample as a whole (*M*=2.86, *SD*=1.46). Excessive daytime sleepiness (scores >10) was reported by 5/23 (22%) parents. Although parent reported child behavioral difficulties were on average within the normal range, scores for three children were in the high range (scores 17-19) and for another three in the very high range (i.e., scores ≥ 20). These scores reflect elevated scores on especially the subscales conduct and emotion. Pro-social behavior was very low for two children.

**Table 1:** Descriptive statistics (Mean, Standard Deviation, Median, Range) for scales used in the study.

	Mean (SD)	Median	Range
***Baseline***			
**Strengths and Difficulties Questionnaire^a^**			
Emotional Problems Subscale Score	2.35 (2.12)	2.00	0 - 7
Conduct Problems Subscale Score	3.26 (2.49)	3.00	0 -10
Hyperactivity Subscale Score	4.48 (2.15)	4.00	0 – 8
Peer Problems Subscale Score	1.35 (1.56)	1.00	0 – 5
Prosocial Subscale Score	7.13 (2.22)	8.00	1 – 10
Total Difficulties Score	11.44 (6.65)	11.00	2 – 25
**Hypoglycemia Fear Survey Total Score^b^**	68.52 (16.16)	70.00	44 - 105
Behavior Subscale Total Score	33.91 (5.58)	35.00	22 – 44
Worry Subscale Total Score	34.61 (12.18)	32.00	20 – 62
**WHO-5 Total Score^c^**	16.78 (4.08)	16.00	8 - 22
**Epworth Sleepiness Scale Total Score^d^**	6.83 (5.38)	6.00	0 - 24

a Subscales are on scale 0-10. Total difficulties score is on scale 0-40. For total scale and all subscales except the prosocial subscale, a higher score denotes more difficulties and fewer strengths. For the prosocial subscale, a higher score denotes more strengths and fewer difficulties.

b Total score is on scale 26-130. Behavior subscale is on scale 11-55, and worry subscale is on scale 15-75. Higher score denotes more fear. Mean score for items on the HFS-B subscale was 3.08 (SD=0.51). Mean score for items on the HFS-W subscale was 2.31 (SD=0.81).

c Scale 0-25. Higher score denotes better well-being.

d Scale 0-24. Higher score denotes more sleepiness.

Parental well-being scores were negatively associated with fear of hypoglycemia and child behavior but positively associated with child age and diabetes duration (see **Table 2**). Parental well-being scores were negatively associated with fear of hypoglycemia total (*r* = - 0.50, *p* <.05) and subscale scores (*r* = - 0.44, *p* <.05 for HFS-Worry and HFS-Behavior) and child behavior (*r* = - 0.45, *p* = .05) but positively associated with child age and diabetes duration (*r* = 0.58, *p* <.01, *r* = 0.60, *p* <.01). All other between-measures correlations were not significant (see **Table 2**).

**Table 2:** Spearman rho Correlation between Parental Well-being, HbA1C, Child Age, Diabetes duration, Hypoglycemia Fear, Child Behavior and Sleepiness (N=23).

	HbA1C	Age	Diabetes Duration	WHO5	HFS-B	HFS-W	HFS-TOT	ESS	SDQ-TOT
HbA1C	1.00	.41	.26	.21	-.38	-.24	-.27	.05	-.11
Age		1.00	.91^***^	.58^**^	-.19	-.24	-.26	-.09	-.22
Diabetes Duration			1.00	.60^**^	-.06	-.13	-.14	-.01	-.24
WHO5				1.00	-.44^*^	-.44^*^	-.50^*^	-.21	-.45^*^
HFS-B					1.00	.63^***^	.81^***^	.22	.22
HFS-W						1.00	.95^***^	.32	.31
HFS-TOT							1.00	.31	.33
ESS								1.00	.13
SDQ-TOT									1.00

*p < .05, **p < .01, ***p < .001.

WHO5, Well-being measure; HFS-B, Hypoglycemia fear behavior; HFS-W, Hypoglycemia fear worry; HFS-TOT, Hypoglycemia fear total; ESS, Epworth sleepiness scale; SDQ-TOT, SDQ Total Score.

## Discussion

Parents of very young children with T1D reported on their worries and behavior in relation to hypoglycemia before commencing a closed-loop intervention. The hypoglycemic fear ratings in the current sample were higher than previously reported for a sample of parents of children with T1D aged 6 and above (5). HFS-worry subscale scores were however comparable to those reported by parents of children younger than 7 years (12). Parents indicated they especially worry about hypoglycemia during the night and will regularly get up to check the blood glucose values in order to prevent hypoglycemia or reduce fear of hypoglycemia. This confirms previous reports, suggesting that nights are particularly difficult for parents of young children with T1D (12).

No relationship was found between daytime sleepiness and other constructs nor with child age or HbA1c. This lack of association suggests that excessive daytime sleepiness in this group of parents is not directly related to the management of their child´s diabetes but could rather be caused by the poorer quality of sleep of the child, general sleep deprivation or irregular sleep patterns (24).

Poor well-being was observed in 2/23 (9%) parents. This percentage is lower than previously reported in parents of youth with diabetes, and may indicate that diabetes and its management affects parental well-being differently depending on the developmental stage of the child (25). The negative association between nocturnal fear of hypoglycemia and perceived well-being suggest that fear of hypoglycemia may be one of the key contributors to the diabetes burden. Parental well-being was positively associated with diabetes duration and child age, whereby child age and diabetes duration were also highly associated. It may be that caring for a very young child is generally challenging for parents and especially so when the child is diagnosed with T1D. As children grow older, parents may adjust to both parenting and diabetes management, even when this does not reduce hypoglycemia fear. Further research is needed to investigate to what extent this relates to general parenting issues or is affected by the specific challenges associated with caring for a very young child with T1D.

Twenty-six percent of the parents (6/23) reported child behavioral difficulties in the high to very high range, signified by 13% (3/23) scoring very high on subscale emotional difficulties and 17% (4/23) on conduct problems. These findings support previous research, indicating that parents often report higher levels of behavioral difficulties for children with T1D, most notably a higher incidence of anxiety and depression and externalizing problems (7, 8, 26).

A negative association was found between parental well-being and child behavior, but not HbA1c levels. Well-being of parents is considered relevant for the long-term outcome in children with diabetes and reduced parental well-being is associated with an increase in child psychosocial problems and worse glycemic control (25, 27, 28). Although our results confirm the association between parental well-being and perceived child behavior, no association with either construct and HbA1c levels was found. This may be related to the child´s age, as the glycemic control in very young children may be mainly determined by the management behavior of the parent/caregiver and less affected by child behavior.

Fear of hypoglycemia was not related to HbA1c levels. This suggests that parental burden is mainly affected by parental anxiety concerning the management of their child´s diabetes rather than the actual glycemic control. To this extent, it is imperative that intervention programs are offered to support parents in a timely manner and, in effect, improve long-term outcomes of glycemic control, child behavior and parental well-being (8, 26).

Some limitations should be considered. Results should be interpreted with caution given the small sample size. Although post-hoc power analyses [using G*Power (29)] indicate that the sample size of N=23 provided at least .74 power to detect effect sizes ≥.44., medium and smaller effects may not have been detected. Furthermore, our data are all based on parent´s reflective reporting. In future studies, observational data or diaries could be used to obtain other indications of parents´ and children´s actual behavior. In addition, the current results are based on cross-sectional data, allowing for the investigations of association. Longitudinal studies are however necessary to investigate causal relationships between the constructs.

Our study also has some specific strength. Using a multicenter and multinational design, our data can confirm that the burden of diabetes in the very young children is universal and needs to be identified and addressed within diabetes care programs to improve long-term diabetes outcomes and prevent psychological/behavioral complications. One option may be the use of hybrid closed-loop systems with age appropriate algorithms, reducing nocturnal risk of hypoglycemia and glucose variability. Such intervention has been found to reduce diabetes burden (15, 30). In addition, family-based interventions, aimed to support parents in managing their child´s diabetes have had positive effects on glycemic control as well as parent-child relationships (26).

## Data Availability Statement

The original contributions presented in the study are included in the article/supplementary material, further inquiries can be directed to the corresponding author.

## Ethics Statement

The studies involving human participants were reviewed and approved by Cambridge East Research Ethics Committee in the UK, the Ethics Committee of the University of Innsbruck in Austria, the Ethics Committee of the University of Vienna in Austria, the Ethics Committee of the University of Graz in Austria, the Ethics Committee of the Medical Faculty of the University of Leipzig in Germany, and the Comité National d’Ethique de Recherche in Luxembourg. Written informed consent to participate in this study was provided by the participants’ legal guardian/next of kin.

## Author Contributions

RH and NA coordinated the study. RH, SH, EF-R, TK, CA, CdB, FC, BR-M, JM, MW and MT designed the study. MT, JA, KN, MFr, JY, EM, CdB, DS, MF, US, AT, DA, SS, SH, EF-R, TK, CA, CB, FC, and BR-M screened and enrolled participants and arranged informed consent from the participants. MT, JA, KN, MFr, JY, EM, CdB, DS, MF, US, AT, DA, JM, HK, SS, SH, EF-R, TK, CA, CdB, FC, and BR-M provided patient care and took samples. JS managed randomization. NC, JS, CdB and IP-tC did or supported data analyses, including the statistical analyses. RH designed and implemented the glucose controller. CdB and IP-tC wrote the manuscript. All authors critically reviewed the report. CdB and IP-tC had full access to all of the data in the study and take responsibility for the integrity of the data and the accuracy of the data analysis. All authors contributed to the article and approved the submitted version.

## Funding

This project has received funding from the European Union’s Horizon 2020 research, Grant/Award Number: 731560; JDRF, Grant/Award Number: 3-SRA-2016-297-M-N; Jaeb Center for Health Research, Grant/Award Number: 3-SRA-2016-297-M-N; European Union’s Horizon 2020 research and innovation programme, Grant/Award Number: 731560; University of Cambridge; University Hospitals of Leicester, National Health Service Trust; Addenbrooke’s Wellcome Trust Clinical Research Facility; Wellcome Trust Strategic.

## Conflict of Interest

MT reports having received speaker honoraria from Minimed Medtronic and Novo Nordisk. MFr has received speaker honoraria from Minimed Medtronic and has served on advisory boards for Eli Lilly. JM is a member in the advisory board of Becton-Dickinson, Boehringer Ingelheim, Eli Lilly, Medtronic and Sanofi, and has received speaker honoraria from ABBOTT Diabetes Care, Astra Zeneca, Eli Lilly, Nintamed, Novo Nordisk, Roche Diabetes Care, Sanofi, Servier and Takeda. MW has received license fees from Becton Dickinson and has served as a consultant to Beckton Dickinson. SH declares speaker honoraria from Eli Lilly and Sanofi. EF-R reports having received speaker honoraria from Minimed Medtronic and Eli Lilly, serving on advisory boards for Eli Lilly. TK has received speaker honoraria from Minimed Medtronic, Roche and Eli Lilly and is member of an advisory board for ABBOTTt Diabetes Care. CdB has received speaker honoraria from Minimed Medtronic and is member of their European Psychology Advisory Board. FC does attend Advisory Boards and obtain speaking fees for ABBOTTt, Medtronic, Lilly, and NovoNordisk. BR-M reports having received speaker honoraria from Minimed Medtronic, Eli Lilly, Roche, Menarini and Novo Nordisk, serving on advisory boards for Eli Lilly. RH reports having received speaker honoraria from Eli Lilly, Dexcom and Novo Nordisk, receiving license fees from Medtronic, and being director at CamDiab.

The remaining authors declare that the research was conducted in the absence of any commercial or financial relationships that could be construed as a potential conflict of interest.

## Publisher’s Note

All claims expressed in this article are solely those of the authors and do not necessarily represent those of their affiliated organizations, or those of the publisher, the editors and the reviewers. Any product that may be evaluated in this article, or claim that may be made by its manufacturer, is not guaranteed or endorsed by the publisher.

## References

[1] DiMeglio LA Acerini CL Codner E Craig ME Hofer SE Pillay K. ISPAD Clinical Practice Consensus Guidelines 2018: Glycemic Control Targets and Glucose Monitoring for Children, Adolescents, and Young Adults With Diabetes. Pediatr Diabetes (2018) 19:105–14. 10.1111/pedi.12737 30058221

[2] Sundberg F Barnard K Cato A de Beaufort C DiMeglio LA Dooley G. Managing Diabetes in Preschool Children. Pediatr Diabetes (2017) 18:499–517. 10.1111/pedi.12554 28726299

[3] Streisand R Swift E Wickmark T Chen R Holmes CS. Pediatric Parenting Stress Among Parents of Children With Type 1 Diabetes: The Role of Self-Efficacy, Responsibility, and Fear. J Pediatr Psychol (2005) 30(6):513–21. 10.1093/jpepsy/jsi076 16055489

[4] Patton SR Dolan LM Smith LB Thomas IH Powers SW. Pediatric Parenting Stress and its Relation to Depressive Symptoms and Fear of Hypoglycemia in Parents of Young Children With Type 1 Diabetes Mellitus. J Clin Psychol Med Settings (2011) 18:345–52. 10.1007/s10880-011-9256-1 PMC3199319 21643962

[5] Viaene AS Van Daele T Bleys D Faust K Massa GG. Fear of Hypoglycemia, Parenting Stress, and Metabolic Control for Children With Type 1 Diabetes and Their Parents. J Clin Psychol Med Settings (2017) 24:74–81. 10.1007/s10880-017-9489-8 28280962

[6] Monaghan MC Hilliard ME Cogen FR Streisand R. Nighttime Caregiving Behaviors Among Parents of Young Children With Type 1 Diabetes: Associations With Illness Characteristics and Parent Functioning. Fam Syst Heal (2009) 27:28–38. 10.1037/a0014770 19630443

[7] Hilliard ME Monaghan M Cogen FR Streisand R. Parent Stress and Child Behaviour Among Young Children With Type 1 Diabetes. Child Care Health Dev (2011) 37:224–32. 10.1111/j.1365-2214.2010.01162.x 21083686

[8] Sweenie R Mackey ER Streisand R. Parent-Child Relationships in Type 1 Diabetes: Associations Among Child Behavior, Parenting Behavior, and Pediatric Parenting Stress. Fam Syst Health (2014) 32:31–42. 10.1037/fsh0000001 24294984 PMC3950351

[9] McDonnell CM Northam EA Donath SM Werther GA Cameron FJ. Hyperglycemia and Externalizing Behavior in Children With Type 1 Diabetes. Diabetes Care (2007) 30:2211–5. 10.2337/dc07-0328 17563334

[10] Jaser SS Whittemore R Ambrosino JM Lindemann E Grey M. Coping and Psychosocial Adjustment in Mothers of Young Children With Type 1 Diabetes. Child Heal Care (2009) 38(2):91–106. 10.1080/02739610902813229 PMC2675938 19412355

[11] Patton SR Dolan LM Henry R Powers SW. Fear of Hypoglycemia in Parents of Young Children With Type 1 Diabetes Mellitus. J Clin Psychol Med Settings (2008) 15:252–9. 10.1007/s10880-008-9123-x PMC2737676 19104970

[12] Van Name MA Hilliard ME Boyle CT Miller KM DeSalvo DJ Anderson BJ. Nighttime is the Worst Time: Parental Fear of Hypoglycemia in Young Children With Type 1 Diabetes. Pediatr Diabetes (2018) 19:114–20. 10.1111/pedi.12525 PMC5650950 28429581

[13] Steineck I Ranjan A Nørgaard K Schmidt S. Sensor-Augmented Insulin Pumps and Hypoglycemia Prevention in Type 1 Diabetes. J Diabetes Sci Technol (2017) 11:50–8. 10.1177/1932296816672689 PMC5375081 28264173

[14] Tauschmann M Allen JM Nagl K Fritsch M Yong J Metcalfe E. Home Use of Day-and-Night Hybrid Closed-Loop Insulin Delivery in Very Young Children: A Multicenter, 3-Week, Randomized Trial. Diabetes Care (2019) 42:594–600. 10.2337/dc18-1881 30692242

[15] Musolino G Dovc K Boughton CK Tauschmann M Allen JM Nagl K. Reduced Burden of Diabetes and Improved Quality of Life: Experiences From Unrestricted Day-and-Night Hybrid Closed-Loop Use in Very Young Children With Type 1 Diabetes. Pediatr Diabetes (2019) 20:794–9. 10.1111/pedi.12872 PMC6771658 31140654

[16] Clarke WL Gonder-Frederick LA Snyder AL Cox DJ. Maternal Fear of Hypoglycemia in Their Children With Insulin Dependent Diabetes Mellitus. J Pediatr Endocrinol Metab (1998) 11(Supplement):189–94. 10.1515/JPEM.1998.11.S1.189 9642659

[17] Topp CW Østergaard SD Søndergaard S Bech P. The WHO-5 Well-Being Index: A Systematic Review of the Literature. Psychother Psychosom (2015) 84:167–76. 10.1159/000376585 25831962

[18] Topp CW Østergaard SD Søndergaard S Bech P. The WHO-5 Well-Being Index: A Systematic Review of the :iterature. Psychother Psychosom (2015) 84:167–76. 10.1159/000376585 25831962

[19] Johns MW. A New Method for Measuring Daytime Sleepiness: The Epworth Sleepiness Scale. Sleep (1991) 14:540–5. 10.1093/sleep/14.6.540 1798888

[20] Goodman R. The Strengths and Difficulties Questionnaire: A Research Note. J Child Psychol Psychiatry Allied Discip (1997) 38:581–6. 10.1111/j.1469-7610.1997.tb01545.x 9255702

[21] Goodman R Scott S. Comparing the Strengths and Difficulties Questionnaire and the Child Behavior Checklist: Is Small Beautiful? J Abnorm Child Psychol (1999) 27:17–24. 10.1023/A:1022658222914 10197403

[22] Goodman R. Using the Strengths and Difficulties Questionnaire (SDQ) to Screen for Child Psychiatric Disorders in a Community Sample. Br J Psychiatry (2000) 177:534–9. 10.1192/bjp.177.6.534 11102329

[23] Theunissen MHC Vogels AGC De Wolff MS Crone MR Reijneveld SA. Comparing Three Short Questionnaires to Detect Psychosocial Problems Among 3 to 4-Year Olds. BMC Pediatr (2015) 15:1–8. 10.1186/s12887-015-0391-y 26178201 PMC4502948

[24] Jaser SS Foster NC Nelson BA Kittelsrud JM DiMeglio LA Quinn M. Sleep in Children With Type 1 Diabetes and Their Parents in the T1D Exchange. Sleep Med (2017) 39:108–15. 10.1016/j.sleep.2017.07.005 PMC7650845 29157581

[25] Eilander MMA Snoek FJ Rotteveel J Aanstoot HJ Bakker-Van Waarde WM Houdijk ECAM. Parental Diabetes Behaviors and Distress Are Related to Glycemic Control in Youth With Type 1 Diabetes: Longitudinal Data From the DINO Study. J Diabetes Res (2017) 2017. 10.1155/2017/1462064 PMC5742467 29376080

[26] Delamater AM de Wit M McDarby V Malik JA Hilliard ME Northam E. ISPAD Clinical Practice Consensus Guidelines 2018: Psychological Care of Children and Adolescents With Type 1 Diabetes. Pediatr Diabetes (2018) 19:237–49. 10.1111/pedi.12736 30058247

[27] Duke DC Geffken GR Lewin AB Williams LB Storch EA Silverstein JH. Glycemic Control in Youth With Type 1 Diabetes: Family Predictors and Mediators. J Pediatr Psychol (2008) 33:719–27. 10.1093/jpepsy/jsn012 18296726

[28] Naar-King S Idalski A Ellis D Frey M Templin T Cunningham PB. Gender Differences in Adherence and Metabolic Control in Urban Youth With Poorly Controlled Type 1 Diabetes: The Mediating Role of Mental Health Symptoms. J Pediatr Psychol (2006) 31:793–802. 10.1093/jpepsy/jsj090 16322274

[29] Faul F Erdfelder E Buchner A Lang A-G. Statistical Power Analyses Using G*Power 3.1: Tests for Correlation and Regression Analyses. Behav Res Methods (2009) 41:1149–60. 10.3758/BRM.41.4.1149 19897823

[30] Ziegler C Liberman A Nimri R Muller I Klemenčič S Bratina N. Reduced Worries of Hypoglycaemia, High Satisfaction, and Increased Perceived Ease of Use After Experiencing Four Nights of MD-Logic Artificial Pancreas at Home (DREAM4). J Diabetes Res (2015) 2article I:1–8. 10.1155/2015/590308 PMC4637058 26581230

